# Evaluating rural household well-being and empowerment among women and young farmers in Senegal

**DOI:** 10.1016/j.dib.2023.109975

**Published:** 2023-12-20

**Authors:** Cyrus Muriithi, Caroline Mwongera, Wuletawu Abera, Christine Chege, Issa Ouedraogo

**Affiliations:** The Alliance of Bioversity International and International Center for Tropical Agriculture (CIAT), Duduville Campus Off Kasarani Road P.O. Box 823-00621, Nairobi, Kenya

**Keywords:** Social economic data, Quasi experiment, Baseline, Sampling

## Abstract

This article provides a description of baseline survey data that was collected in Senegal in the regions of Sedhiou and Tambacounda in 2020, respectively, and as part of an agricultural development project aimed at improving the well-being and resilience of farming households. The survey was implemented using a structured questionnaire administered among 1503 households, 70% of whom are women and 30% are young people, in the two regions. This paper contains data that can helps in understanding the socioeconomic well-being and resilience of smallholder farming households, especially among women and youth. This data helps to associate information on: (i) the socioeconomic project area variables, (ii) the extent of use of irrigated and climate change-adapted crops; (iii) the level of soil and water resource management in the study regions; and (iv) the food security and dietary diversity with the well-being and empowerment of women and young smallholder farming households. In addition, the dataset can be used as a baseline or reference point to track the economic empowerment and climate resilience building achieved in the study regions.

Specification Table

**Table:** 

Subject	Smallholder farming households’ women and youth empowerment via agricultural capacity building
Specific subject area	Regions of Sedhiou and Tambacounda in Senegal
Type of data	Quantitative and qualitative data (Categorical, numeric, and string variables) presented as tables, graph, and figures.
How the data were acquired	Face to face interviews were conducted using a structured questionnaire at household level.
Data format	Raw dataset in Stata file format. Analyzed
Description of data collection	For data collection, a baseline study was conducted to evaluate the project's implementation. The study focused on women (35+ years) and young farmers (18-34 years) actively engaged in farming activities.
Data source location	Data collection occurred in two regions of Senegal: Sedhiou and Tambacounda. In the Sedhiou region, data collection was conducted in three departments: Sedhiou, Bounkiling, and Goudomp. In the Tambacounda region, data collection took place in four departments: Tambacounda, Goudiry, Bakel, and Koumpentoum.
Data accessibility	The data in this paper will be fully made public at the end of fifth year of the project period as required by the project's data sharing policy. This will be in Havard Data Verse. For this publication, the data can be accessed here: https://doi.org/10.7910/DVN/DV0RLJ
Related research article	The paper related to this data has been published already at Heliyon Journal.

## 1 Value of the Data

• The data highlights a significant knowledge gap about women and youth farming practices in the prevailing climate change conditions (droughts due to increase in temperature) by providing an overview of the primary constraints for socioeconomic empowerment, climate resilience building, and sustainable development in Sedhiou and Tambacounda especially among women and youths.
• The data may be used by research and development partners to better understand the social and cultural dynamics of conducting a household baseline survey targeting women in Senegal, particularly in the Sedhiou and Tambacounda regions.
• This baseline survey data may support policy outreach efforts and inform targeted development strategies aimed at smallholder farming households, with a focus on women and youth empowerment.
• This baseline survey data can support program implementers by informing the development of tailored and adaptive agricultural interventions for smallholder farming households, particularly programs targeting women and youth.
• The dataset may be used to analyze how different Climate Smart Agricultural (CSA) practices effect agricultural productivity in rural farming households.
• The dataset is useful for analyzing socioeconomic, institutional, and cultural factors that may impact smallholder farming households’ adoption of CSA practices and may participate in certain dynamics affecting women and youths.
• The baseline datasets contain rich information to contribute to generation of resilience index which is the ultimate goal of the project in the performance management framework. The data can be grouped into major resilience pillars of assets, access to basic services, adaptive capacity, and social safety nets.
• In the realm of academia, particularly within the field of statistics, this dataset offers a chance to explore the application of Propensity Score Matching (PSM) methods in quasi-experimental approach. It also provides insights into addressing and rectifying biases associated with this approach.

## 2 Objective

The dataset was generated through a baseline survey conducted before the start of field operations. The survey aimed to capture a snapshot of the baseline conditions in the area where the project activities will take place. Specifically, the household survey was utilized to gather essential socioeconomic information on project locations and beneficiaries. This dataset is essential for providing a comprehensive understanding of the area's socioeconomic conditions and the project's potential impact on the beneficiaries.

## 3 Data Description

The baseline survey data collection instrument was designed in accordance with the Adaptation and Valorization of Entrepreneurship in Irrigated Agriculture (AVENIR) project performance management framework. The International Center for Tropical Agriculture (CIAT) performed a baseline study in the Sedhiou and Tambacounda regions from October to December 2020 in conjunction with the Institut Sénégalais de Recherches Agricoles (ISRA). Conducting the baseline survey prior to the start of field operations provides a snapshot of the baseline conditions in the area where the project activities will be carried out. The household survey was used to collect critical socioeconomic information on project locations and beneficiaries. As such, the survey data contains information on social and economic attributes, farming operations, common responses to climate change and shocks, social groupings, nutrition knowledge and habits, as well as the availability and use of extension services.

Small-holder farmers were sampled from each department in the study regions: the Sedhiou region, encompassing Bounkiling, Goudomp, and Sedhiou departments; and the Tambacounda region, encompassing Bakel Koumpentoum, Goudiry, and Tambacounda departments. Proportionate multistage sampling was used to account for the number of administrative units (areas, departments, communes, and arrondissements) in each of the two research regions. The baseline survey was designed in an open data kit (ODK) hosted by surveyCTO, which improved efficiency and security in collecting data from remote places in both research zones.

We define a household as a person or group of individuals who live in the same house or complex, share the same housekeeping arrangements, and are cared for as one unit, which means they make common food provision and routinely eat from the same pot [7]. The household head (chef de ménage) is considered the ultimate decision-maker on land use and other household characteristics; Hence, prerequisite to contacting specific farming household members who fit the inclusion criteria, data collectors would ensure that the household head (chef de ménage) gave their permission to the respondent to participate in the survey.

As shown in Table 1, the directory contains the following data. There are two versions of the data: an R-dataset and an Excel version. A PARENT KEY in the child dataset or a KEY in the mother dataset is the connection between the two datasets. You can download the survey codebook for more details about each variable in the datasets.

**Table 1:** Description of the files in the directory.

File name (. xlsx, .Rds)	Contents
Agro-agrop	Details about agro processing of selected crop by the household
borrowfrom-credit	Details about household's experience with borrowing money or other items (in-kind)
Clean Full Baseline dat	Master dataset (all-in-one) file
crop_p2	Details about seed sources for selected crops
crop_p3	Details about fertilizer for the selected crops
Csa	Details about agricultural practices done by the households
Dome	Details about domestic assets owned by the households
Ext	Details about provider and sources of extension Advice
Farm	Details about farm assets owned by the households
foodI	Details about food groups consumed by the household in the past 7 days
income_q444	Details about other (apart from agriculture) different sources for an household
Irri	Details about crops irrigated by the household in the past 1 year and the methods used
lasset_own	Details about plot ownership and characteristics (farm details)
lasset_ownb	Details about plot ownership and characteristics (labour details)
Lives	Details about lives assets owned by the households
market_infos_market	Details about marketing details of the harvested crop products
NFE1	Details about purchase of non-food items and other expenditure for domestic consumption
NFE2	Details about the amount of money spent on non-food items purchased
Prob	Details about the shocks that can be linked to food scarcity and seasonality
problems_remit	Details about household internal and international migration and remittances
Proc	Details of specific agro processing activity on selected crop by the household
product_inform	Details on sale of harvested crop products by the households
q611	Details about irrigation method and source of water
q930	Details about amount of money spent on goods and services in the past 30 days
q2505	Details about the seed inputs in terms of quantity, price and variety
q2524	Details about the fertilizer in terms of quantity, price
q2530_p4	Details about harvest from tree by a household in the past 1 year
Roster	Details about household roster characteristics
Shock	Details about climate and shock experienced by the households
socialG	Details about social groups that households normally participate in.
Trans	Transport assets owned by the households
Tree	Details about types of trees that household grows in their plots

## 4 Experimental Design, Materials, and Methods

### 4.1 Study site

The AVENIR project's effect evaluation strategy uses a quasi-experimental approach due to the purposeful selection of program beneficiaries (treatment) and non-beneficiary (control) target areas. Boukiling and Goudomp were chosen as treatment departments in the Sedhiou region, whereas Sedhiou was chosen as the control department in that region. Tambacounda and Goudiry were chosen as treatment departments in the Tambacounda region, whereas Bakel and Koumpentoum were chosen as the control departments.

### 4.2 Sampling strategy

The primary focus of the baseline survey was the household. We utilized a multistage sampling approach to identify survey respondents. The project specifically targets rural arrondissements, which are subdivided into villages, each composed of multiple households.

**Fig. 1:**
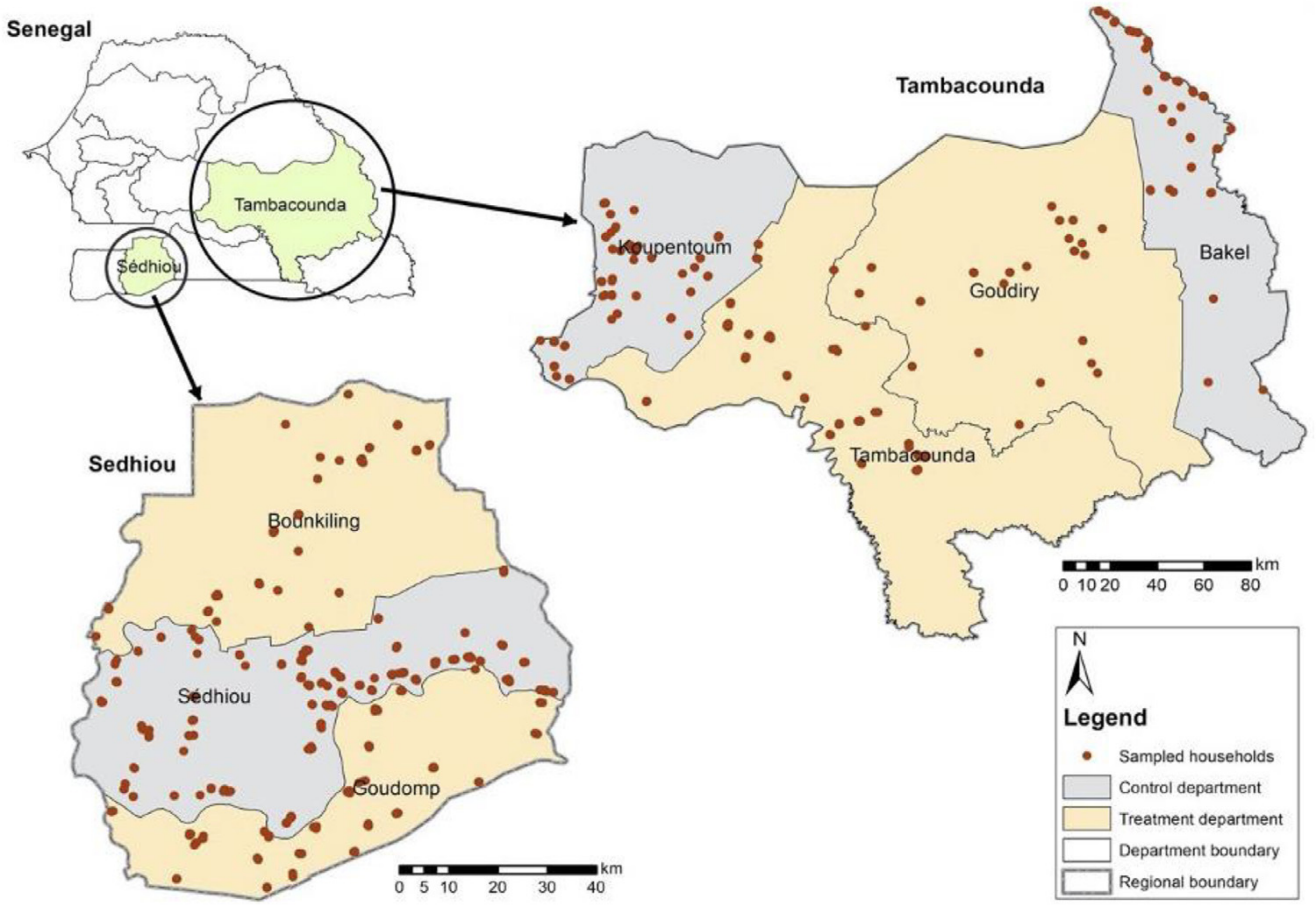
Map of project sites showing the locations of sampled households in the baseline study. source [6].

The key inclusion requirements for participation in the baseline survey included being a smallholder farmer from the Sedhiou and Tambacounda region and being either female over the age of 35 years or a young person between the ages of 18-34. Fig. 1 depicts the research areas where data was collected in Tambacounda and Sedhiou.

During the analysis, PSM is used to select similar treatments and control households (analysis sample) based on key observable household characteristics. A machine learning feature selection technique was used to select these household characteristics, which included household size, total domestic assets, number of irrigated crops, number of tree varieties grown, number of income sources, number of good agricultural practices used, and membership in social groups or multi-stakeholder platforms.

### 4.3 Sample size determination

We used a sample size calculation for comparative studies paired with Cochran's sampling framework [2] to establish the number of samples necessary to detect a difference between two proportions for all the indicators examined at baseline level in the AVENIR project.

$$n={\left(Z_{\alpha /2}+Z_{\beta }\right)}^{2}*\left(p_{1}\left(1-p_{1}\right)+p_{2}\left(1-p_{2}\right)\right)/{\left(p_{1}-p_{2}\right)}^{2},$$

Where:

• Z_α/2_ is the critical value of the Normal distribution at α/2 (e.g., for a confidence level of 95%, α is 0.05 and the critical value is 1.96),
• Z_β_ is the critical value of the Normal distribution at β (e.g., for a power of 80%, β is 0.2 and the critical value is 0.84) and
• p_1_ and p_2_ are the expected sample proportions of the two groups.

A sample size of 1503 agricultural families were calculated using this method for both control and treatment departments in the Sedhiou and Tambacounda regions. The villages in the region were identified using the Agence nationale de la Statistique et de la Démographie – (ANSD) 2013 general census of people, housing, agriculture, and livestock [8]. This sampling framework directed our two-stage sampling strategy. The advantage of this strategy is that it is time-efficient while still being a dependable data collection strategy.

During the initial multi-stage sampling at the village level, villages were selected with unequal probability with a discount proportional to village size in each stratum (department). The total number of households in each village determined the assumed community size. The following equation describes the likelihood of selecting a village being included in a study:

$$P_{1hi}=\frac{n_{h}*m_{hi}}{M_{h}}\;where\;M_{h}=\sum m_{hi}$$

Where:

$P_{1hi}$ is the probability of selecting Village i in stratum h in the first degree;

$n_{h}$: The number of Villages to be drawn in stratum h;

$m_{hi}$: The number of households in Village i in stratum h;

$m_{hi}$: The number of households in stratum h.

In the second sampling phase, the number of villages to be drawn in each department was computed using the weights of the different strata based on the beneficiary and control groups. The distribution of communities chosen in each department stratum is shown in Table 2.

**Table 2:** Sample of beneficiary and control villages by region and department.

	Sedhiou Region				Tambacounda Region				
Departments	Sédhiou	Goudomp	Boukiling	Total	Tambacounda	Goudiry	Bakel	Koumpentoum	Total
Treatment villages (%)	-	25 (53%)	22 (47%)	47	24 (51%)	23 (49%)	-	-	47
Control villages (%)	60 (100%)	-	-	60	-	-	26(43%)	35 (57%)	61
Total	-	-	-	107	-	-	-	-	108

Note: Figures in parentheses reflect the proportion of villages drawn from each department

A consistent number of 7 households were randomly picked in each village selected in the first sampling phase, for both the treatment and control villages guided by sampling with equal probability method. Lists of active farming households submitted by village leaders created a sample frame for the second stage sampling at the household level (equal chance draws).

As part of the project requirement of reaching out to 70% women and 30% youth, the seven households picked in each village were selected as follows: five households with at least one woman over the age of 35, and two households with at least one young person between the ages of 18 and 35. These selection minima was defined after gathering information from the village leader. The data collection enumerators performed random draws with equal possibilities (for example, tossing a coin) to minimize selection biases. If multiple people were eligible to be surveyed at household level, another draw was held in each selected home. Table 3 shows the representative sample at the department level.

**Table 3:** Household sampled by village per departments.

Departments	Total Village	Household per village	Total households
Sedhiou	60	7	420
Goudomp	25	7	175
Boukiling	22	7	154
Tambacounda	24	7	167
Goudiry	23	7	161
Koumpentoum	35	7	244
Bakel	26	7	182
Total	**215**	Avg = 7	**1503**

### 4.4 Survey administration

The questionnaire was designed and hosted on Android tablets using the open data kit (ODK) specifically surveyCTO platform. A team of forty enumerators with data gathering experience was chosen with the assistance of an implementation partner (ISRA). In each of the two regions, two senior supervisors were assigned to oversee data collection efforts. Another two supervisors were chosen locally within the departments to assist with daily monitoring and logistical coordination.

Prior to data collection, the enumerators were trained for a week and the survey questions were translated into the French and local language (wolof). Based on the local sociocultural norms, eligible participant households were identified through the village heads *(chefs de village* in French), who provided lists of productive farming households in the communities. The enumerators carried out on average one-and-a-half-hour face-to-face interviews with the targeted and consenting respondents. At the household level, consent was obtained from the household leader (*chef de menage* in French), who is considered the principal decision maker in a homestead. Typically, the household head makes the final decision on the agricultural operations carried out by the households in their homestead. In their absence, another individual is often considered to be the primary decision maker in the homestead.

A homestead is comprised of numerous households based on the local residential structure. A parent, for example, may have his sons (married or unmarried) residing in the same compound..

**Fig. 2:**
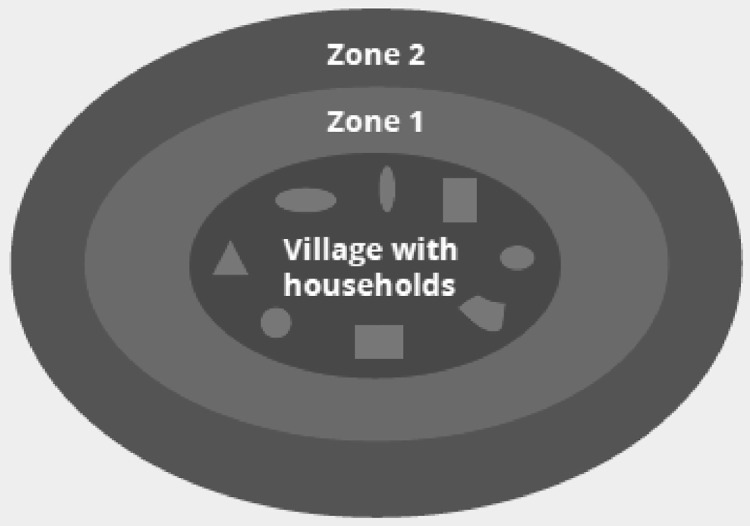
Land use zones in Sedhiou and Tambacounda region source [6].

Farming households can lease or acquire more farmland inside or outside of their community. In zone 1 (Fig. 2) for instance, each household is allocated a plot of farmed land for cereals and other uses. In the outer zone 2, each household has a larger plot of land for growing cash crops.

Among the variables assessed were demographic factors, social factors, on-farm and off-farm socioeconomic factors, agro-processing attributes, climatic and irrigation factors, income and remittances, extension services, as well as sanitation and nutrition. We will use the baseline data in this article to estimate and quantify the effect of various interventions against the midline and endline data to demonstrate changes over time and to monitor progress. Using screening criteria and defined sampling methodology, target respondents were randomly selected. As part of the project's data sharing policy, this baseline data will be shared publicly on the Harvard data verse.

### 4.5 Questionnaire modules

The general goal of the questionnaire was to determine the baseline level conditions of the two regions and among women and young farmers. It also aimed at defining a current status of empowerment for women and youth in the Sedhiou and Tambacounda regions.

1. **Household characteristics**

Information regarding the target respondent's socioeconomic status (SES), household characteristics, and household members’ characteristics was collected.

2. **Household assets.**

Under this module, we gathered data on each household's mobile and immovable assets in order to create a normal wealth index proxy, a domestic asset index, an asset index discrepancy, and a housing quality index.

3. **Land use**

In this module, surveyors inquired about the number of agricultural plots each household owns and uses for farming; access and land ownership; labor (resources required to manage agricultural plots); crop tree farming, harvesting, and marketing; crop production and management; and market and agro-processing of products.

4. **Household income sources, financial services and credit**

We collected information on the various nt revenue sources and its use by the household, the amount income generated, and the principal decision-maker for income utilization. Data was also collected on the households’ access to various financial services and sources of credit.

5. **Migration and remittances**

We collected data on migration for participants who reported having worked beyond the household, region and country bounderies. Data was collected on reasons for migrating, the respondant's levels of schooling, curent employment status, remittance habits, and remittance use, if any.

6. **Climate variability and shocks**

This section asked about the types of climate information services that respondents had accessed in the previous five years, how they used this information, the impact of rainfall and temperature changes impacts on agricultural activities in the past ten years the types of climate shocks that the household had experienced in the past ten years, and any action taken by the household to manage climate shocks.

7. **Irrigation and climate smart agriculture (CSA)**

We the respondents’ use of irrigation and agricultural technologie, the types of crop grown under irrigation on their farms, type of irrigation system used, and their willingness to pay for irrigation water services.

8. **Social network**

This module looked at household engagement in various social groups by evaluating group membership, farmers' contributions to group decision-making, farmers' influence and position in the group, and the type of help the farmer received from the group in the previous year. We asked farmers who are members of an irrigation and water user association (IWUA) about water-related disputes or conflicts; the number of yearly disputes and if they were resolved; and who was involved in resolving the problem; and the current status of the disagreement.

9. **Sanitation and nutrition**

We inquired about the major source of drinking water, the time it takes to get to the water source, water treatment, the kind of toilet facility, and waste disposal.
The food consumption collected data on household dietary diversity, food intake, quantity of food consumed, amount of money spent on food and non-food items in the family, coping strategies during periods of food insecurity, and assessment of food insecurity.
The food consumption questions relate to twelve food groups (WFP, [9]; [5] that were consumed by households in the previous seven days. The Food Consumption Scores (FCS) reflects the quality and quantity of food available at the household level and may be used to determine the prevalence of various levels of food insecurity by categorizing families as poor, borderline, or satisfactory. The greater the nutritional diversity and frequency of food consumption, the higher the food consumption score. A high food consumption score thus translates a higher chance of nutritional adequacy in a family.
The food expenditure share (FES) module aims to quantify household economic vulnerability and is used as an indication of household food security. It's is calculated by dividing household food spending by total food and non-food expenditure during a thirty-day period. The greater the proportion of household income spent on food, the more exposed the household is to food insecurity [3].
We wanted to measure dietary quality and quantity at the household level with the Household Dietary Diversity Score (HDDS) [5]. The HDDS questions in this study were centered on twelve dietary groups: cereals, white tubers and roots, legumes, nuts and seeds, vegetables, fruits, meat, eggs, fish and fish products, milk and milk products, sweets and sugars, oils and fats, spices, condiments, and drinks [4]. A family with a higher HDDS is thought to have a healthier diet than one with a lower HDDS.
We employed a series of questions that reflect general domains and sub-domains of experiencing household food insecurity with a recall time of thirty days for the home food insecurity access scale (HFIAS). It focused on insufficient food access. The HFIAS indicator was created for this purpose, and based on earlier research [1,5]. The HFIAS values range from 0 to 27, with zero indicating that the household is food secure and 27 indicating extreme food insecurity.
The children/infant module was designed for the household's youngest kid under the age of five. We began by gathering health information on the reference kid from the mother or caregiver. In addition, we inquired about newborn dietary variety as prescribed by the World Food Program (WFP) and the World Health Organization (WHO). The women's dietary diversity asked for the reference woman's health information, and the minimal dietary diversity (MDD-W), where we were able to assess the reference woman's micronutrient sufficiency.

10. **Extension services**

In this module, we asked about access to various services, such as providers of extension services and barriers to access of such services,; how farmers adopted advice from various service providers; and the distance between the farmer and the service provider.

11. **Covid-19**

In this module, we asked about the impact of COVID-19 on the overall well-being of the household, as well as the household's coping strategies in the face of the pandemic.

## Ethical Statement

The questionnaire was developed in accordance with the project logical framework with inputs from all project partners. CIAT obtained internal authorization (#2019-IRB30) from its ethical approval body while our key implementing partner ISRA has an ethical clearance from Senegal government in conducting agricultural research within Senegal.

## Credit Statement

We thank Katiana Bougouma, amd Derek Linzey (Scriptoria Solutions) for the content review and copyediting of the manuscript.

## Data Availability

AVENIR Baseline Study (Original data) (Dataverse).
